# Fall risk classification for people with lower extremity amputations using random forests and smartphone sensor features from a 6-minute walk test

**DOI:** 10.1371/journal.pone.0247574

**Published:** 2021-04-26

**Authors:** Kyle J. F. Daines, Natalie Baddour, Helena Burger, Andrej Bavec, Edward D. Lemaire

**Affiliations:** 1 Ottawa Hospital Research Institute, Ottawa, Ontario, Canada; 2 Department of Mechanical Engineering, University of Ottawa, Ottawa, Canada; 3 University Rehabilitation Institute, University of Ljubljana, Ljubljana, Slovenia; 4 Faculty of Medicine, University of Ljubljana, Ljubljana, Slovenia; West Park Healthcare Centre, CANADA

## Abstract

Fall-risk classification is a challenging but necessary task to enable the recommendation of preventative programs for individuals identified at risk for falling. Existing research has primarily focused on older adults, with no predictive fall-risk models for lower limb amputees, despite their greater likelihood of fall-risk than older adults. In this study, 89 amputees with varying degrees of lower limb amputation were asked if they had fallen in the past 6 months. Those who reported at least one fall were considered a fall risk. Each participant performed a 6 minute walk test (6MWT) with an Android smartphone placed in a holder located on the back of the pelvis. A fall-risk classification method was developed using data from sensors within the smartphone. The Ottawa Hospital Rehabilitation Center Walk Test app captured accelerometer and gyroscope data during the 6MWT. From this data, foot strikes were identified, and 248 features were extracted from the collection of steps. Steps were segmented into turn and straight walking, and four different data sets were created: turn steps, straightaway steps, straightaway and turn steps, and all steps. From these, three feature selection techniques (correlation-based feature selection, relief F, and extra trees classifier ensemble) were used to eliminate redundant or ineffective features. Each feature subset was tested with a random forest classifier and optimized for the best number of trees. The best model used turn data, with three features selected by Correlation-based feature selection (CFS), and used 500 trees in a random forest classifier. The resulting metrics were 81.3% accuracy, 57.2% sensitivity, 94.9% specificity, a Matthews correlation coefficient of 0.587, and an F1 score of 0.83. Since the outcomes are comparable to metrics achieved by existing clinical tests, the classifier may be viable for use in clinical practice.

## Introduction

Falling is the second leading cause of accidental injury death [1]. Injuries related to falling can be debilitating, life-altering, and lead to lower self-perceived balance confidence [2]. Although preventative programs exist to reduce the chance of falling such as muscle strengthening, life-style changes, and Tai Chi [3], the initial task of fall-risk identification can be challenging. While more than 26 fall risk assessment tools are available for clinicians [4], evolving wearable sensors systems can provide opportunities to augment common clinical mobility tests for fall risk identification.

Wearable technology has been used to develop fall-risk classifiers that provide accurate and automated fall risk classification, to enable timely intervention with fall-risk mitigation techniques. Previous research has used a variety of sensors and technology, such as inertial sensors, for fall risk classification [5–10]. Features can be extracted from data collected during clinical tests such as the 6 minute walk test (6MWT) [5] or 10 meter walk test [6]. The 6MWT includes straight and turn walking, measures functional capacity, and is widely used to measure the response of therapeutic interventions [11]. Recently, a study with stroke survivors reported a moderate relationship with 6MWT and fall risk [12]. Research reports differences in turning strategies between fall-risk and no fall-risk populations, and that viable elderly fall-risk classification can be achieved with turn data [5,9]. If fall risk classification can be determined from the 6MWT, clinic time could be more efficiently used by reducing the number of tests in a session.

Previous studies have used sensors at multiple locations on the body [5,6]. This can be problematic in the clinic due to the time required to setup sensors on the person, sensors falling off or sliding, and patients changing how they move due to too many sensors, and accuracy of their position. Therefore, the number of sensors should be minimized, and sensors should have easy and reliable placement on the body. For example, the pelvis was reported as the best single sensor location for fall-risk classification [6], and the most commonly used location [13]. One technique for collecting pelvis data is to use The Ottawa Hospital Rehabilitation Center (TOHRC) Walk Test app [14]. This app allows for a low cost and easily accessible option to have multiple sensors in one device at a single sensor location.

The fall-risk classification literature has focused primarily on elderly populations, with no predictive fall risk models for lower limb amputees. However, lower limb amputees are at higher risk of falling compared to able-bodied and other clinical populations in all phases of rehabilitation [15,16], and the risk of fall related injuries requiring medical care can be higher than older adults [17]. Predictive models developed for able-bodied or elderly populations do not necessarily translate to the lower limb amputee population. The purpose of this research was to determine the best features and models to perform fall-risk classification in amputees who are completing a 6MWT and ensure that similar results can be achieved when compared to existing assessment tools.

## Materials and methods

The work was approved by the Ethic Committee of the University Rehabilitation Institute, Slovenia. It was approved on April 18th, 2018 (approval number No 46/2018), and re-approved for an additional 30 participants on September 9th, 2019 (No. 27/2019). Written consent was obtained by each participant.

### Participants

A convenience sample of 129 participants with lower limb amputations were recruited from the University Rehabilitation Institute (Ljubljana, Slovenia) and gave informed consent for this study. Clinical records provided self-reported number of falls, with falling at least once in the past six months prior to testing considered fall risk. For this study, data from 89 participants (19 female, 70 male, age 62.3 ±12.5) were suitable for fall risk classification (32 fall-risk, 57 no fall-risk). Participants included 4 bilateral transtibial amputees, 1 bilateral transtibial and transfemoral, 63 transtibial, 18 transfemoral, 2 knee disarticulation, and 1 ankle disarticulation, with an average time since amputation of 15.6±17.3 years. Some participants indicated when falling most often occurred: 2 said not while wearing their prosthesis, 4 said equally often with and without the prosthesis, 18 said while wearing the prosthesis, but the prosthesis is not the cause, 7 said falling due to the prosthesis, and 58 said the cause was unknown. Inclusion criteria were ankle disarticulation or higher amputation of at least one lower limb, walking with a prosthesis for at least 6 months, functional prosthesis (good socket fit, not broken), and willing to participate. Exclusion criteria were wounds on stump or other foot that may worsen with 6MWT, all other medical conditions that will be contraindicated for 6MWT, and a broken prosthesis. Reasons for unsuitable data were malfunctions exporting data from phone (5 people), no fall incidence data on file (9 people), running instead of walking during the 6MWT (1 person), or had unidentifiable foot strikes due to highly irregular gait (2 people), a single crutch (2 people), double crutches (18 people), or non-rolling walker (3 people). Participants using canes or walkers who had identifiable foot strikes were included.

### Equipment

**Fig 1:**
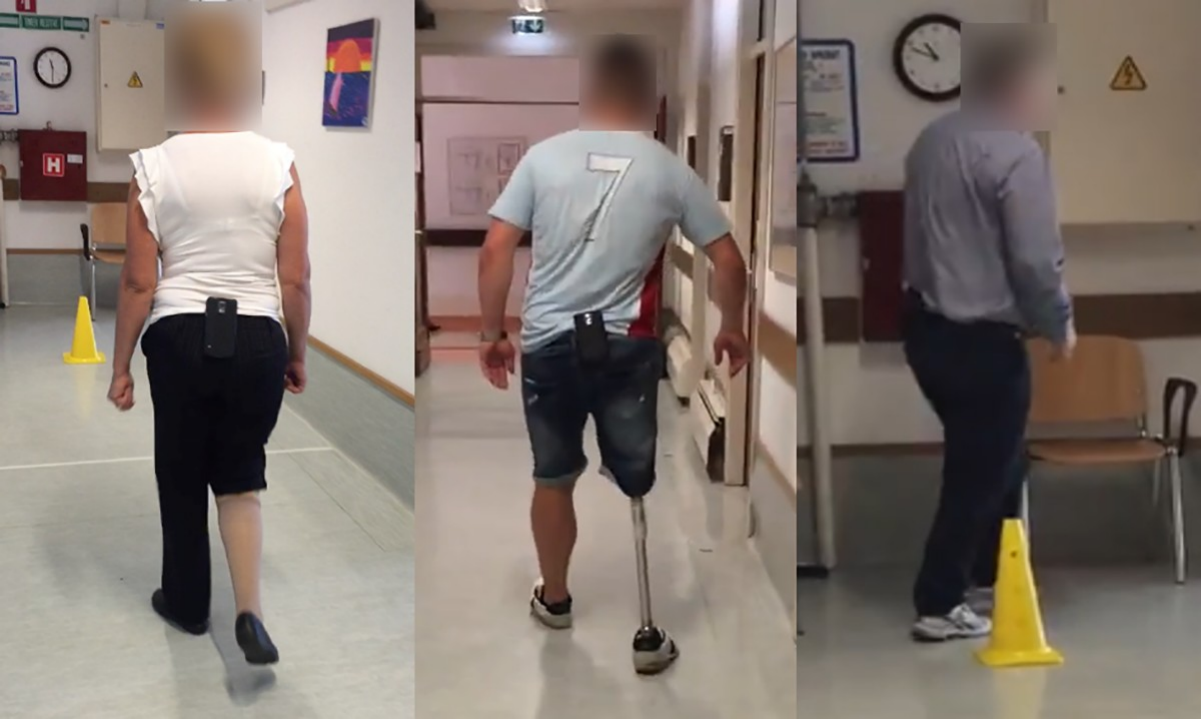
6MWT with smartphone on posterior pelvis.

**Fig 2:**
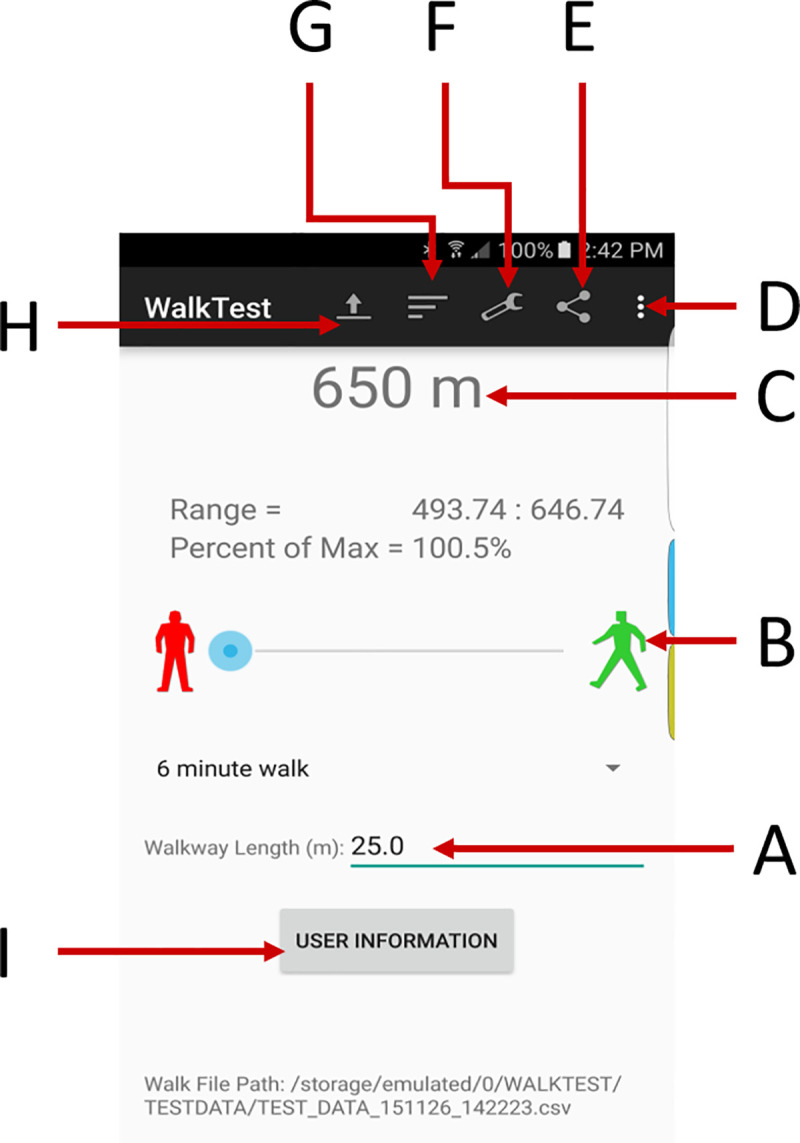
TOHRC walk test application. (A) Walkway length; (B) Start trial, (C) Distance walked; (D) About, Help; (E) Share output; (F) Settings; (G) Results tables; (H) Load previous data; (I) Enter patient demographics.

An Android smartphone was affixed to the midline of the posterior pelvis using a waist belt (Fig 1). Participant demographics and information were input into a custom designed The Ottawa Hospital Rehabilitation Center (TOHRC) Walk Test app [14] (Fig 2). Each participant performed a 6MWT along a 20m hallway (i.e., walk, turn around a cone, and continue the circuit for 6 minutes). The TOHRC Walk Test app collected smartphone 3D accelerometer and gyroscope raw data, and pelvic rotation, tilt, and obliquity at 50 Hz. Each trial was also video recorded. Once the test was complete, data were exported from the smartphone to a text file for post-processing.

### Step and turn segmentation

**Fig 3:**
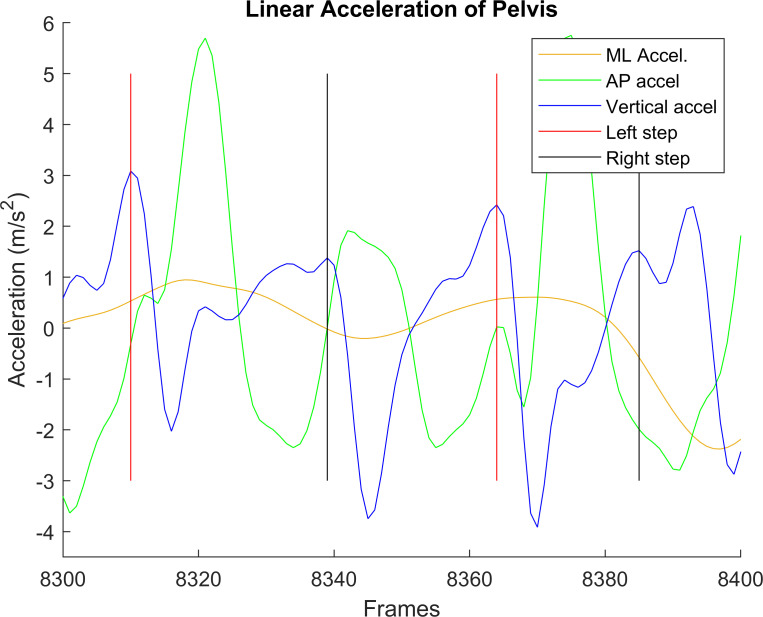
**Anterior-posterior (blue), vertical (green), and medial-lateral (yellow) accelerations before manual step identification.** Black lines indicate right steps, red lines indicate left steps. Manual step identification was required around frame 8340.

**Fig 4:**
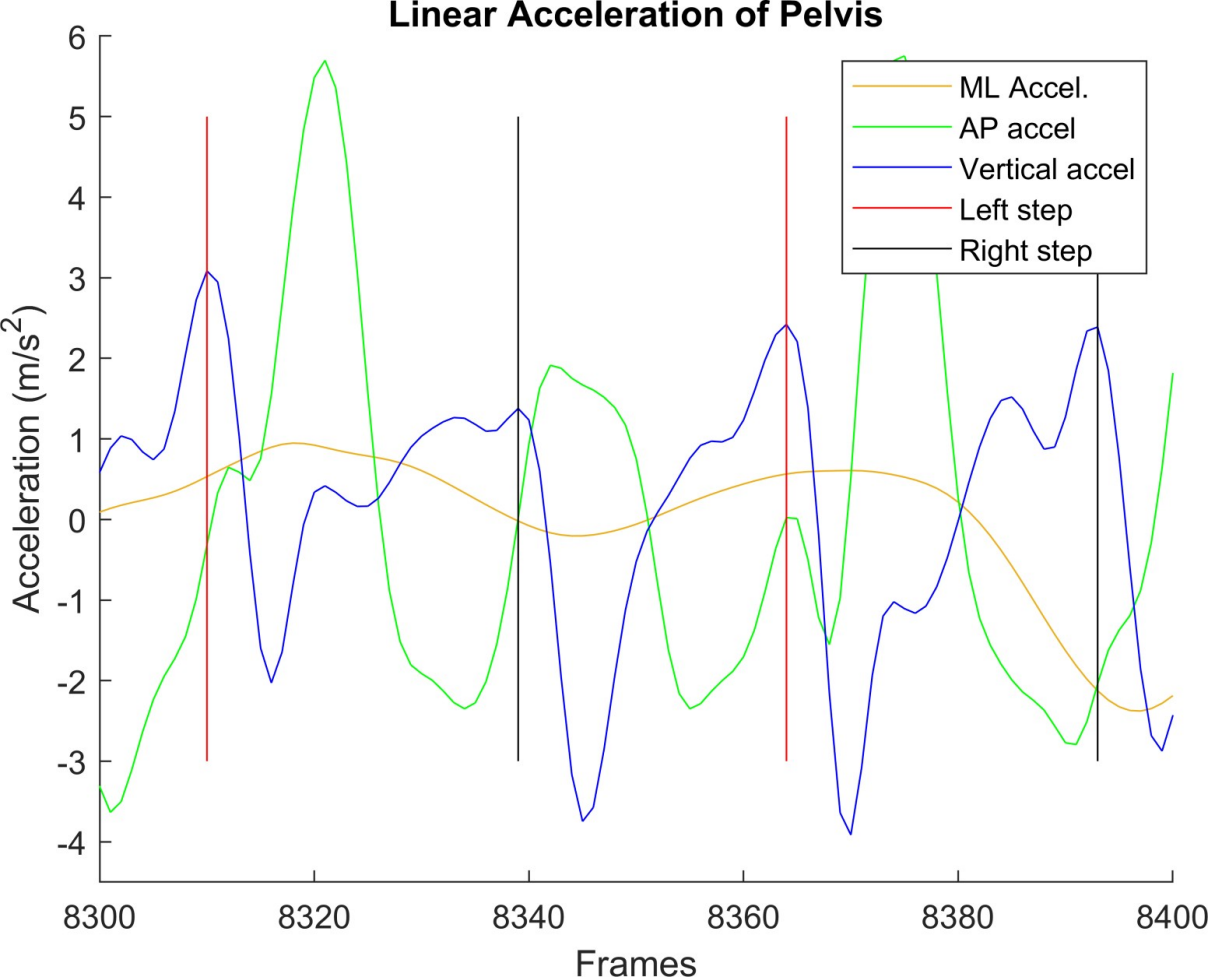
**Anterior-posterior (blue), vertical (green), and medial-lateral (yellow) accelerations after manual step identification.** Black lines indicate right steps, red lines indicate left steps.

Raw accelerometer data, gyroscope data, and smartphone orientation were imported to MATLAB 2013b, along with the time stamps for each recording. Foot strikes were identified from anterior-posterior (AP) linear acceleration (Figs 3 and 4), using the peak value near the estimated next step, based on average step duration [14]. AP acceleration had the least variance when compared to other linear acceleration axes. However, this automated technique for able-bodied gait [14] sometimes failed to select the correct peak with lower limb amputees due to amputee participant’s more asymmetric and variable gait (e.g., Fig 3). In these cases, manual step identification was required (Fig 4). The manually cleaned data was used to extract features.

In previous research, data from turn walking was better at classifying fall risk than data from straight walking [5]. Therefore, data were segmented into turns and straightaways. Differences between straight and turn steps (Fig 4) include greater medial-lateral accelerations and a greater anterior-posterior acceleration peak in the middle of the turn. Turns were defined as the five steps around the center of each turn. The center of a turn was identified using pelvis rotation, by using the middle frame between the beginning and end of pelvis rotation (i.e., when the pelvis started to rotate and when it stopped rotating). Two steps before and two steps after this middle step constituted the five steps. Straightaways were all other steps. Similar to foot strikes, this process was first automated in MATLAB, then verified manually.

Once turn and straightaway steps were identified, four feature sets were created. The first feature set calculated features for all steps (AS) without distinguishing between straightaway and turn steps. The second and third feature sets were straightaway (S) and turn (T) step feature sets. The fourth feature set was the combination of the S and T feature sets (S&T), therefore doubling the number of features, but keeping the distinction between the two types of steps.

### Feature extraction

Based on existing literature [5,13], features were extracted from linear acceleration and angular velocity signals in each step. 62 features were extracted for the four feature sets:

#### Temporal

Cadence, step time (foot strike to foot strike of the opposite foot), stride time (foot strike to foot strike of the same foot), symmetry in right and left limb step times (symmetry index) [18].

#### Descriptive statistics

Minimum, maximum, mean, standard deviation, root mean square in three axes (vertical, medial lateral (ML), AP) for pelvis linear acceleration (Android processed signal, not including gravity) and tilt, rotation, and obliquity angular velocities.

#### Linear acceleration and angular velocity frequency domain features

From the absolute value of the Fast Fourier transform (FFT) of each step, the First Quartile of Fourier transform (FQFFT), Ratio of Even/Odd Harmonics (REOH), and peak distinction.

#### FQFFT

Percentage of frequencies within the first quartile of the Nyquist frequency (6.25 Hz was used as the first quartile). Lower FQFFT values indicate more high frequency components, linked to instability [19].

#### REOH

Ratio of the frequencies in the even harmonics compared to the odd harmonics (using stride time as the fundamental frequency). Lower REOH values have been associated with fall risk [5,20].

#### Peak distinction

**Fig 5:**
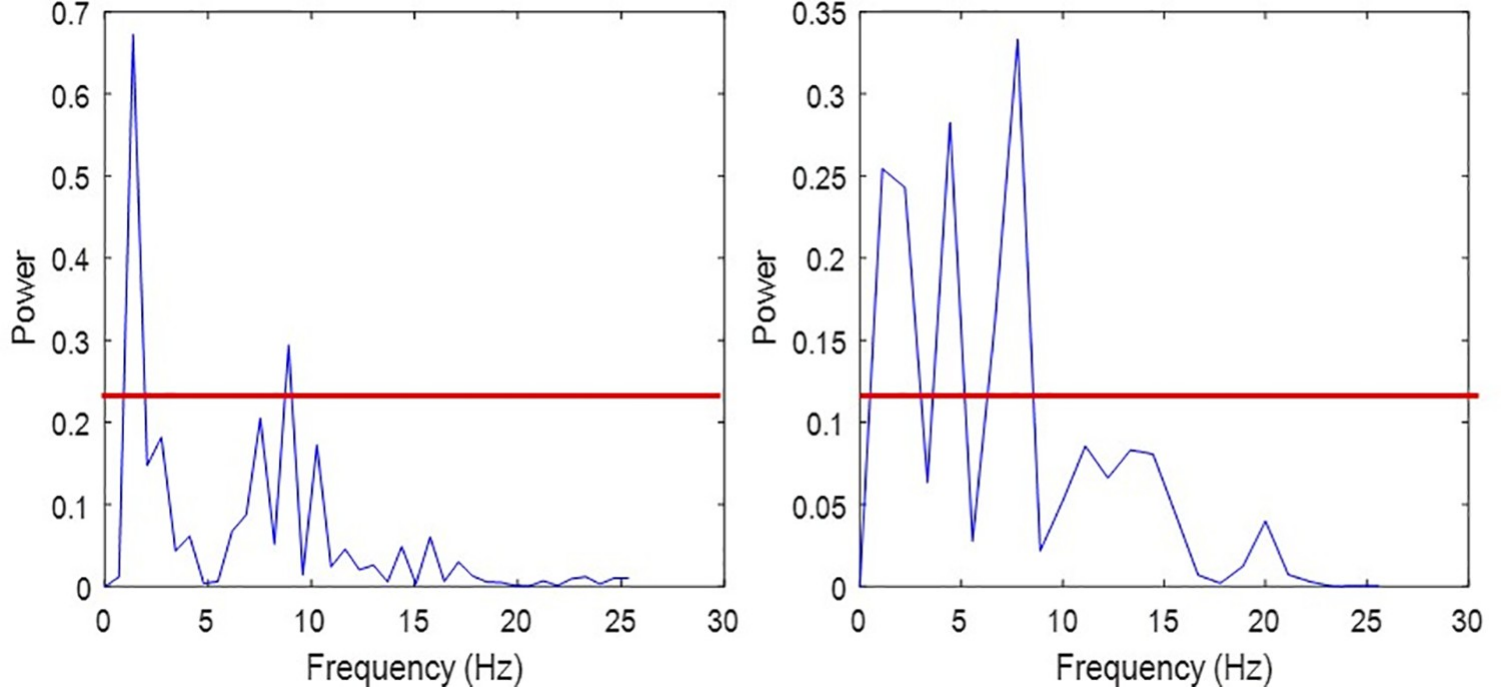
Peak distinction for two different steps. Peak distinction for two steps. (a) A step with a very distinct peak, having a peak distinction of 5.26%. (b) A step with a less distinct peak, having a peak distinction of 20.83%. Horizontal red lines indicate one third of max amplitude.

To determine if the FFT peak frequency was distinct from other frequencies, the percent of frequencies in the FFT with power greater than a threshold (⅓ amplitude of peak signal) was calculated. A lower peak distinction value means a more distinct peak (Fig 5).

Once features were extracted for each step, the minimum, maximum, mean, and standard deviation were calculated over all included steps for a total of 248 features (62 multiplied by 4 statistics) per data set (496 for the S&T data set).

### Feature selection

Feature selection was used to reduce feature space dimensionality, simplifying the problem by removing redundant and irrelevant data [5,6,21]. Three feature selection techniques were used, based on previous success in fall risk classification: Correlation-based feature selection (CFS) [22], Relief-F (RelF) [23,24], and an extra trees classifier ensemble method (ETC) [5,25].

CFS is a supervised, filter-based method that identifies a subset of features that are correlated with the class label (i.e., fall-risk or no fall-risk), but also uncorrelated to other parameters by calculating the “merit” based on pair-wise correlations [22]. This allows CFS to develop a subset that has no irrelevant or redundant features, by only adding features that improve the subset’s merit.

RelF is a supervised method that ranks features by weighting them based on their relevance and how well instances from different classes and the same class can be distinguished [23,26]. RelF does not eliminate redundant features, making this method most useful when evaluating parameters with interdependencies.

ETC is an ensemble method that fits a number of randomized decision trees on various sub-samples of the dataset and uses averaging to improve the predictive accuracy and control over-fitting [25]. Each feature is then ordered based on their importance and the best features can be selected. An ETC ensemble method was used in this research because, while they are very similar to random forests, ETC computes using randomly selected weightings, making ETC better for feature selection [27].

For RelF and ETC, features sets were created for the top 30, 20, 10, and 5 features. CFS selected less than 5 features for all sets.

### Classification techniques and optimization

A random forest classifier with 100 trees and a leave-one-out strategy was applied to the data set. Random forests consist of many decision trees operating as an ensemble, making them preferable to a single decision tree. Features are sets of variables calculated from the sensor signals, which are used as input for the decision trees so that the model can learn to predict a sample’s class. A random forest classifier takes a majority vote across multiple trees to decide which class the model should predict [28]. The belief is that the trees are uncorrelated so that operating as a committee allows them to outperform any of the individual models.

Five evaluation metrics were used for evaluating the models: accuracy, sensitivity, specificity, Matthews correlation coefficient, and F1 score. The best five “feature selector–data set combinations” were chosen based on a ranking technique similar to [6] and [29]. Each classifier was ranked in the five evaluation metrics, and the lowest summed rankings were chosen as the top five classifiers, with five being the lowest possible summed ranking since five metrics were used. These five models were then optimized for the number of trees that provided the highest accuracy by testing increments from 5 to 1000 trees. More trees perform better with minimal risk of overfitting, although more trees increase computation times [27]. To test robustness, each of the five optimized models were built 10 times with different random seeds in a leave-one-out strategy to determine the mean and standard deviation for accuracy, sensitivity, specificity, and MCC score.

## Results

The mean result for the 6MWT was 289.8m ± 118.0m.

### Feature selection

Each feature selector chose different features, creating a range of subsets (Tables 1 and 2). For turn data, CFS chose only three features but provided the best overall feature set (vertical acceleration maximum standard deviation, AP acceleration minimum peak distinction, and tilt angular velocity minimum peak distinction). Straight walking CFS only chose one feature, standard deviation of vertical acceleration’s standard deviation. S&T and AS CFS chose combinations of these 4 features, so that all CFS subsets selected similar features.

**Table 1:** Legend of feature descriptions for feature sets in Table 2.

Variable	Statistic (Feature #)	Variable	Statistic (Feature #)
Maximum Vertical acceleration	Max (10), Std Dev (12)	FQFFT tilt angular velocity	Mean (119)
Maximum AP acceleration	Mean (15), Std Dev (16)	FQFFT rotation angular velocity	Min (121)
Mean ML acceleration	Std Dev (32)	Maximum of tilt angular velocity FFT	Mean (143)
Mean Vertical acceleration	Min (33)	Standard deviation of ML acceleration FFT	Min (153)
Standard deviation of vertical acceleration	Max (46), Std dev (48)	Standard deviation of AP acceleration FFT	Min (161)
Tilt angular velocity range	Std Dev (56)	Standard deviation of rotation angular velocity FFT	Mean (171)
Tilt angular velocity mean	Min (65)	Standard deviation of obliquity angular velocity FFT	Std Dev (176)
Rotation angular velocity mean	Std Dev (72)	Peak distinction of ML acceleration	Mean (179)
Obliquity angular velocity mean	Mean (75)	Peak distinction of vertical acceleration	Max (182), Mean (183)
Step timing	Max (90), Mean (91), Std Dev (92)	Peak distinction of AP acceleration	Min (185)
Stride timing	Min (97), Max (98), Mean (99), Std Dev (100)	Peak distinction of tilt angular velocity	Min (189)
Cadence	Min (101), Mean (103)	Peak distinction of rotation angular velocity	Min (193), Max (194)
FQFFT ML acceleration	Min (105), Std Dev (108)	Peak distinction of obliquity angular velocity	Mean (199)
FQFFT vertical acceleration	Std Dev (112)	REOH of vertical acceleration	Min (205), Max (206), Mean (207)
FQFFT AP acceleration	Mean (114)	Root Mean Square of Rotational Angular Velocity	Min (241)

**Table 2:** Top 30 selected features for each feature selector.

T-CFS	T-RelF	T-ETC	S-CFS	S-RelF	S-ETC	S&T-CFS	S&T-RelF	S&T-ETC	AS-CFS	AS-RelF	AS-ETC
46	100	65	48	90	171	48 (S)	100 (T)	193 (S)	46	98	75
185	90	207		92	103	46 (T)	90 (S)	98 (S)	48	189	101
189	91	98		56	121	185 (T)	90 (T)	183 (S)	185	90	32
	92	15		16	205	189 (T)	56 (S)	179 (T)	189	99	199
	207	176		12	108		91 (T)	121 (S)		92	194
	98	100		99	48		91 (S)	153 (T)		97	10
	72	103		183	182		12 (S)	100 (T)		56	241
	185	119		91	105		185 (T)	32 (S)		91	33
	97	185		97	161		98 (T)	153 (S)		185	143
	206	72		101	16		99 (S)	97 (T)		101	114

T = Turn, S = Straight, S&T = Straight and Turn, AS = All steps. Refer to Table 1 for feature descriptions.

AP linear acceleration minimum peak distinction was the only feature selected in the top ten by all three feature selectors, for turn data. More fall-risk participants had lower peak distinction for minimum AP linear acceleration and tilt angular velocity, meaning that more fall-risk participants had distinct FFT peaks.

### Model optimization

Table 3 shows the unoptimized results for fall risk classification using each feature selector and a random forest classifier with 100 trees.

**Table 3:** Unoptimized ranked metrics for top 10 subsets with a random forest classifier. Numbers after feature selector ETC and RelF indicate the number of features from that subset.

Feature Selector	Accuracy (%)	Sensitivity (%)	Specificity (%)	MCC	F1	SR
T-CFS	78.7	53.1	93.0	0.521	0.642	6
S&T-CFS	74.2	53.1	86.0	0.417	0.596	16.5
S-ETC10	70.8	43.8	86.0	0.331	0.519	24.5
S-RelF10	70.8	40.6	87.7	0.326	0.500	27
AS-RelF30	69.7	40.6	86.0	0.301	0.491	37.5
AS-ETC30	69.7	40.6	86.0	0.301	0.491	37.5
T-ETC30	69.7	37.5	87.7	0.295	0.471	45
ASCFS	68.5	50.0	78.9	0.299	0.533	54.5
S&T-RelF10	67.4	46.9	78.9	0.270	0.508	63.5
StT-RelF30	68.5	34.4	87.7	0.264	0.440	65

T = Turn, S = Straight, S&T = Straight and Turn, AS = All steps, MCC = Matthews Correlation Coefficient, F1 = F1 score, SR = summed ranking.

The top five models were rebuilt using different numbers of trees, ranging from 5 to 1000. Results for the best model (T-CFS) are shown in Table 4. Table 5 provides a summary for all the best trees.

**Table 4:** Tree optimization for best model (T-CFS).

T-CFS	Accuracy (%)	Sensitivity (%)	Specificity (%)	MCC	F1
5	76.4	46.9	93.0	0.467	0.588
10	79.8	59.4	91.2	0.547	0.679
25	71.9	46.9	86.0	0.360	0.545
50	75.3	53.1	87.7	0.442	0.607
75	77.5	53.1	91.2	0.493	0.630
100	78.7	53.1	93.0	0.521	0.642
150	79.8	59.4	91.2	0.547	0.679
200	79.8	50.0	96.5	0.555	0.640
250	80.9	59.4	93.0	0.574	0.691
300	79.8	56.3	93.0	0.548	0.667
400	82.0	56.3	96.5	0.606	0.692
500	82.0	56.3	96.5	0.606	0.692
1000	80.9	56.3	94.7	0.576	0.679

T = Turn, MCC = Matthews Correlation Coefficient, F1 = F1 score.

**Table 5:** Summary of tree optimizations for the 5 best models.

Feature Selector	Trees	Accuracy (%)	Sensitivity (%)	Specificity (%)	MCC	F1
T-CFS	500	82.0	56.3	96.5	0.61	0.69
S&T-CFS	500	80.9	56.3	94.7	0.58	0.69
S-ETC10	250	71.9	46.9	86.0	0.36	0.55
S-RelF10	50	71.9	50.0	84.2	0.37	0.56
AS-RelF30	150	70.8	50.0	82.5	0.34	0.55

T = Turn, S = Straight, S&T = Straight and Turn, AS = All steps, MCC = Matthews Correlation Coefficient, F1 = F1 score.

Once the optimal number of trees was determined, ten models with different random seeds were built for each model to determine robustness (Table 6).

**Table 6:** Final mean and standard deviation (in brackets) metrics for optimized models based on 10 random seeds.

Feature Selector	Accuracy (%)	Sensitivity (%)	Specificity (%)	MCC	F1
T-CFS	81.3 (1.09)	57.2 (2.11)	94.9 (1.29)	0.59 (0.027)	0.69 (0.019)
S&T-CFS	78.9 (1.38)	55 (1.61)	92.3 (1.48)	0.53 (0.033)	0.65 (0.021)
S-ETC10	69.3 (1.5)	41.6 (3.31)	84.9 (0.91)	0.30 (0.038)	0.49 (0.032)
S-RelF10	68.7 (2.4)	44.7 (4.43)	82.1 (3.07)	0.29 (0.055)	0.51 (0.039)
AS-RelF30	69 (1.42)	43.4 (3.74)	83.3 (1.49)	0.29 (0.036)	0.50 (0.031)

T = Turn, S = Straight, S&T = Straight and Turn, AS = All steps, MCC = Matthews Correlation Coefficient, F1 = F1 score.

## Discussion

This research demonstrated that a random forest classifier with smartphone sensor data collected at the posterior pelvis can provide viable fall-risk classification for lower extremity amputees that completed a 6MWT. The best model had 81.3% accuracy, 57.2% sensitivity, and 94.9% specificity. The very high specificity showed that the model had a low chance of false positives, indicating that if the model has a low chance of inappropriately classifying a person as a faller. This almost 95% specificity was higher than other clinical tests that focus on fall risk in amputees [30]. This is important for health and long-term care systems where appropriate resource allocation is essential.

More than half the people with amputations who are at risk of falling would be properly identified (i.e., 57.2% sensitivity), which is an interesting result considering that the 6MWT was not designed as a fall risk measure. However, this sensitivity was lower than other clinical fall risk tests. Two common clinical tools are the Four Square Step Test (FSST) and Timed Up and Go (TUG). In one study predicting multiple falling (2 or more falls in 6 months), FSST with lower limb amputees had a predictive sensitivity of 92% and specificity of 93% in amputees using a cut-off time of 24 seconds, and TUG had a predictive sensitivity of 85% and specificity of 74% [30]. However, the sensitivity and specificity could have been high due to the 2 or more falls criteria for fallers, since this group may have had consistently poorer TUG performance than people who have only fallen once. Instead, a review of fall risk assessments found that the Time-up and Go (TUG) test has a predictive sensitivity of 76% and specificity of 49% on older adults [4]. A study that used wearable sensors with a TUG test achieved a mean sensitivity of 77.3%, and a mean specificity of 75.9% in older adults [9], demonstrating an improvement when wearable sensors were included.

The specificity of the best model from this study was more than 20% greater than the specificity of the Timed Up-and-Go (TUG) test on older adults both with and without wearable sensors. However, best model sensitivity was around 57%; therefore, the 6MWT approach cannot be considered as a surrogate for other fall risk tests. However, people can confidently be classified as fall-risk in a clinic using a 6MWT approach without requiring additional testing, due to the test’s high specificity. In many amputee clinics, specific fall risk test may not be routinely performed, so this 6MWT approach would be useful for identifying other people who may be at risk. If clinicians believe that the individual is a fall-risk, and the person was identified as “no fall risk” from the 6MWT, other clinical fall-risk tests can be performed as indicated.

Random Forest classifiers using feature-selectors have been effective in previous studies on older adults. Using these techniques on an amputee population’s pelvis sensor data from a 6MWT provided similarly effective outcomes for fall risk classification. A previous study on older adults who completed a 6MWT with accelerometers located at the pelvis and ankles achieved 73.4% accuracy, 60.5% sensitivity, and 82.0% specificity [5]. The outcomes from this study agreed with previous work that turn data was better for fall-risk identification, but the models generated for amputee participants had a higher specificity and accuracy. It was important to examine older adults and people with amputations separately since gait patterns differ between these groups and amputee populations have a higher fall risk than older adult populations.

While previous studies typically used multiple sensor locations (e.g., accelerometers at pelvis and shanks for older adults [5]), this research only use smartphone sensors at the posterior pelvis. A single pelvis location provides an approach that is efficient to apply and easily repeatable in the clinic. The proposed smartphone-based method could have better chance of knowledge translation at the point of patient contact. Fall risk classification results could be provided to the clinician immediately following the test by including the model in the smartphone 6MWT application, thereby supporting clinical decision-making with instant reporting. Since the machine learning model uses a random forest classifier, fall-risk classification can be performed rapidly on a mobile device and is achievable now with appropriate smartphone software development and improved automated step detection in amputees.

### Features

Feature selection generally improved classification results since sets with no feature selection were in the bottom 20%. CFS provided noticeably better results than other feature selection techniques, except with straight data where only one feature was selected. Both RelF30 and ETC30 achieved accuracies above 65% for all data sets, and these feature sets were ranked in the top half of all models. However, four of the top five feature subsets had ten or fewer features. While most feature subsets achieved good specificities, smaller feature subsets also had good sensitivities. Smaller subsets may have led to less data overfitting and therefore better fall-risk classification.

The most selected feature for turn data was AP acceleration minimum peak distinction (i.e., most distinct FFT peak). For AP acceleration, if the FFT had one predominant frequency, the peak would have been more distinct since one peak FFT amplitude would have a noticeably greater than the others. Participants who were a fall-risk were more likely to have more distinct peaks.

T-CFS model had the best classification results and included two minimum peak distinctions and one maximum standard deviation. The second-best model (S&T-CFS) used the same features as T-CFS, but also included straight walking vertical acceleration’s standard deviation of the standard deviation. Interestingly, including this single feature decreased all outcome metrics by over 2%.

Turning while walking can be more challenging for people with mobility disabilities, so it is intuitive that a model using turn data provided the most successful classifier. This is consistent with results from an elderly population [5]. Therefore, turn steps should be used for 6MWT-based faller classification. In future research, additional features such as personal health information and the results of the 6MWT could be included. This study only included results obtainable from smartphone sensors.

### Models

The best classification model was T-CFS, closely followed by S&T-CFS. Model performances were also similar to clinical functional assessment tools [4], making the 6MWT smartphone approach a good tool for clinical fall-risk identification. Since most models had relatively high specificity, the sensitivity results contributed most to the overall ranking. This demonstrates the importance of having multiple types of evaluation metrics. Metrics such as accuracy can be inflated due to class imbalance (this data set had 36% of the participants identified as fall-risk). Class imbalance is an unavoidable problem with fall-risk classification since less of the population is at risk of falling. Therefore, classifiers that are better at dealing with slight class imbalances, such as a random forest, should be considered for fall risk classification.

Initial testing with 100 trees resulted in the T-CFS and S&T-CFS performing better than other classifiers. Optimization by adding more trees improved results up to a plateau in effectiveness around 100 or 200 trees. No additional improvements occurred after 500 trees. After the optimal number of trees was selected for each classifier, this optimal number of trees was tested ten times each to verify that the results were robust. Mean results for both CFS models by the end of optimization were better than unoptimized models.

A limitation of this study was that fall-risk was based on individual retrospective self-report, where the response depends on personal recall. Future research could include prospective follow-up questionnaires that provide more details on falls and stumbles. Additionally, the model identifies people who have fallen at least once, which could relate to a different risk of injury when compared to multiple fallers, and therefore different preventative techniques may be required.

Another limitation of this study was that all participants were recruited from only one rehabilitation institute. Future research could consider participants from a variety of countries and clinics. Additionally, increasing the number of participants would help improve the model’s generalizability and possibly improve model effectiveness.

The smartphone app requires that foot strikes be automatically identified, using sensor data from the posterior pelvis location. The current rule-based method appropriately identifies foot strike for free walking and single cane gait; however, foot strike detection errors were found when people used a walker, two forearm crutches, or shuffled their feet (i.e., not foot strike or foot off). Therefore, further research on foot strike detection for these conditions is required before using the app for these three conditions. This could involve new AI foot strike detection models or adding the option of securing IMU sensors to the shoes specifically for these conditions.

Since the best model (T-CFS) had better specificity than TUG in older adults [4] and a sensitivity of 57%, it is reasonable to use T-CFS as a preliminary indicator for fall risk that identifies those who may have previously been missed as fall-risk individuals. This approach could help reduce the number of tests required for a complete functional assessment, since 6MWT are often performed during clinical evaluations and TUG may not be collected for people who can walk for 6 minutes. As more participants are added to the training set, this Random Forest Classifier approach should continue to improve and complement existing functional assessment tools to assist with fall-risk classification. Random Forest Classifier models are computationally efficient and could easily be implemented and run on a mobile device and integrated into a 6MWT app, making the fall risk model accessible for clinicians.

## Conclusions

A novel smartphone sensor-based fall-risk classification method was developed to provide a sensor-based fall-risk classification for lower limb amputees. The best classification model used correlation-based feature selection on turn step features in combination with a random forest classifier. This model had very high specificity, leading to few false negatives. This is important so that patients are not mistakenly suggested into preventative programs. While 57% sensitivity indicated that more than half the people at risk of falling were appropriately classified, future research should aim to improve model sensitivity to identify more people at risk of falling. Turn steps have been found to be the best indicator of fall-risk in both lower limb amputees and older adults, making them the best choice for fall-risk identification. Addition of a single straightaway step feature negatively affected the turn step classifier’s results. The methods developed here for collecting data and classifying individuals can be easily implemented into clinical practice, making it a potential method to indicate a need for fall risk assessment tools. By achieving fall-risk assessment during a 6MWT, the number of required functional mobility tests can be reduced, thereby reducing patient time in clinics.

Future work should continue to add more participants to the dataset, improving the classification metrics to ensure success in clinical implementation. If the larger dataset includes sufficient instances of both single and multiple fallers, research could be initiated to differentiate between these two fall classes. A fall risk model could also be trained and evaluated for a two-minute walk test.
